# MiR-129-5p sensitization of lung cancer cells to etoposide-induced apoptosis by reducing YWHAB

**DOI:** 10.7150/jca.35410

**Published:** 2020-01-01

**Authors:** Chengshan Xu, Zhongli Du, Simei Ren, Xiaoshuan Liang, Huihui Li

**Affiliations:** 1 National Center for Clinical Laboratories, Beijing Hospital, National Center of Gerontology; Institute of GeriatricMedicine, Chinese Academy of Medical Sciences, Beijing, China; 2 Department of Breast Surgery, Harbin Medical University Cancer Hospital, Harbin, China; 3 Department of Medical Oncology, Shandong Cancer Hospital and Institute, Shandong First Medical University and Shandong Academy of Medical Sciences, Jinan, Shandong Province, China

**Keywords:** Lung cancer, miR-129-5p, YWHAB, Apoptosis

## Abstract

**Background:** Lung cancer is the most common cause of death from cancer worldwide and recent studies have revealed that microRNAs play critical roles to regulate lung carcinogenesis. microRNA-129-5p (miR-129-5p) has been reported to regulate cell proliferation and invasion in lung cancer, but its role in lung cancer apoptosis remains unknown.

**Methods:** The expression of miR-129-5p and YWHAB in lung cancer tissues were analyzed from data downloaded from the NCBI Gene Expression Omnibus (GEO) database. Luciferase reporter assay, Western blot and qRT-PCR were used to determine the regulatory effect of miR-129-5p on YWHAB. Cell apoptosis was detected by using the PI/Annexin V Cell Apoptosis Kit. The effect of miR-129-5p and YWHAB on the survival of lung cancer patients was also explored.

**Results:** In this study, by combining the data derived from six GEO database, our results showed that miR-129-5p was downregulated in lung cancer tissues and YWHAB was upregulated in lung cancer patient' serum. A significant negative correlation between miR-129-5p and YWHAB was found in lung cancer tissues. Both the expression of YWHAB and miR-129-5p were associated significantly with prognosis (overall survival) in patients with lung cancer. Overexpression of miR-129-5p promotes VP16-induced lung cancer cell apoptosis and YWHAB was shown to be a direct downstream target of miR-129-5p.

**Conclusion:** Overexpression of expression miR-129-5p contributes to etoposide-induced lung cancer apoptosis by modulating YWHAB.

## Introduction

Lung cancer is the leading cause of cancer-related death worldwide, accounting for more than 1.8 million new cases and almost 1.6 million estimated deaths in 2012 1. In China, lung cancer is the most commonly diagnosed cancer and the leading cause of cancer-related death, which cause about 6 million deaths in 2015 2. Lung cancers are broadly classified into two types: non-small-cell lung cancer (NSCLC) and small-cell lung cancer; NSCLC accounts for the majority of all lung cancer cases, which comprise three major histological subtypes: adenocarcinoma, squamous cell carcinoma and large-cell carcinoma 3.

microRNAs (miRNAs), a kind of small endogenous noncoding RNAs, have been clearly demonstrated to suppress target messenger RNA (mRNA) expression post-transcriptionally by base-pairing with 3'-untranslated region (3'-UTR) of their targets. Differential expression of miRNAs has been revealed between lung cancer and normal tissues and is involved in the progression of lung cancer 4-6.

Aberrant expression of miR-129 has been detected in various types of human cancers and the validated target genes are involved in cancer-related biological processes such as cell proliferation, apoptosis, cell cycle, and metastasis 7-11. Accumulating evidence indicates that miR-129 could play a dual role in tumorigenesis. miR-129 plays a role as tumor suppressors with decreased expression in various tumors 12-16. However, the role of miR-129 in tumorigenesis remains largely elusive 17.

YWHAB encodes a number of 14-3-3 family proteins, which regulates signal transduction by binding to specific Ser/Thr-phosphorylated motifs containing proteins. 14-3-3β, which is encoded by the YWHAB, regulates multiple signaling pathways in normal and cancer cells. 14-3-3β suppressed Cyclin D1 expression and repressed the apoptotic activity through binding phosphorylated SRPK2 and inhibiting its nuclear translocation 18. 14-3-3β decreased bone formation and inhibits osteoblastogenesis in mesenchymal stem cells trough interact with Ror2 19. Sirt2 deacetylated and down-regulated the transcriptional activity of p53, and 14-3-3β augmented the function of Sirt2 in an AKT-dependent manner 20. 14-3-3β increased glucocorticoid receptor transcriptional activity and hepatic gluconeogenesis 21. Down regulation of 14-3-3β decreased p-ERK levels and induced senescence phenotypes in glioblastoma cells 22. Knockdown of 14-3-3β decreased cell viability but increased the LDH release in human glioma U87 cells through inducing ER stress 23. 14-3-3β was upregualted in hepatocellular carcinoma and enhanced liver cancer cell migration and proliferation 24, 25.

## Materials and methods

### Cell culture

A549 and NCI-H1299 (H1299) cell lines purchased from the National Platform of Experimental Cell Resources for Sci-Tech (Beijing, China). Both A549 and H1299 cells were cultured in Dulbecco's modified Eagle's medium with 10% fetal bovine serum, 100 IU/mL penicillin, and 100 μg/mL streptomycin at 37°C in a humidified 5% CO_2_ atmosphere. The medium was changed at alternate days and the cells were split before they reached 100% confluency.

### miRNA mimics, inhibitors, plasmids and cell transfections

miR-129-5p mimics and miR-129-5p inhibitors were synthesized by Genepharma group (Shanghai, China). The full-length 3'UTR of YWHAB was subcloned into the pIS0 luciferase plasmid to generate pIS0-YWHAB-3'UTR 26. Mutant construct of YWHAB-3'UTR, named pIS0-YWHAB-3'UTR-m, which carried a substitution of three nucleotides within the core binding sites of YWHAB-3'UTR, was conducted using mutant PCR primers. Primers used in this study are shown in Supplementary Table 1. Lipofectamine 2000 (Life Technologies Corporation, Grand Island, NY, USA) was used for transfection of DNA plasmids and oligonucleotides according to the manufacturer's protocol.

### Quantitative real time-PCR (qRT-PCR)

We used TRIzol reagent (Life Technologies Corporation) to extract total RNA from the cultured cells. Total RNA was used to synthesize cDNA with a cDNA Synthesis Kit (FastQuant RT Kit, TianGen, Beijing, China). qRT-PCR was performed using SuperReal PreMix Plus (TianGen) according to the manufacturers' recommendations. The sequences of all primers used for qRT-PCR are listed in Supplementary Table 1. All experiments were performed in triplicate. β-actin and U6 expression were used for normalization of the expression of mRNA and MiR-129-5p, respectively. Relative expression levels were determined using the 2^-ΔΔCt^ method.

### Western blotting

Total proteins were extracted using lysis buffer (50 mM Tris-HCl (pH 7.4), 150 mM NaCl, 1% Nonidet P-40, 0.5% sodium deoxycholate, 50 mM NaF, 0.01 mM Na_3_VO_4_) containing phosphatase and protease inhibitors (11873580001, Roche Applied Science). Proteins were separated by sodium dodecyl sulfate-polyacrylamide gel electrophoresis (SDS-PAGE) and then transferred onto polyvinylidene fluoride membranes (Millipore, Bedford, MA, USA). After blocked with 5% bovine serum albumin, the membranes were incubated with the appropriate antibody overnight at 4°C. The antibodies against YWHAB (Abcam, Cambridge, MA, USA, ab97273) and GAPDH (Abcam, ab8245) were respectively used to detect their targeting proteins. The density of the bands was analyzed by using Image J software (NIH Image, Bethesda, MD) and normalized by GAPDH as indicated.

### Luciferase reporter assay

A549 and H1299 cells were cotransfected with pIS0-YWHAB-3'UTR or pIS0-YWHAB-3'UTR-m and miR-129-5p mimics, 48h after transfection, firefly and Renilla luciferase activities were measured according to the Dual-Luciferase Reporter Assay protocol (Promega, Madison, WI, USA). The Renilla luciferase activities were used to normalize transfection efficiency. Experiments were performed in triplicate.

### Flow cytometry

Cells transfected with negative control or miR-129-5p mimics/inhibtors for 24 h were starved overnight and then treated with VP16 (Sigma-Aldrich) for 48 h. The FCM assay was performed using the Annexin V FITC Apoptosis Detection Kit (BD Biosciences, San Jose, CA, USA) according to the manufacturer's instructions.

### Statistical analysis

Data are expressed as the mean ± standard deviation (SD). Data were analyzed by Student's t-test or analysis of variance using SPSS v.17.0 software (SPSS Inc., Chicago, IL, USA). The statistical significance is defined as P < 0.05 (two-sided).

## Results

### miR-129-5p is down-regulated in human lung cancer tissues and associated with lung cancer progression

**Figure 1:**
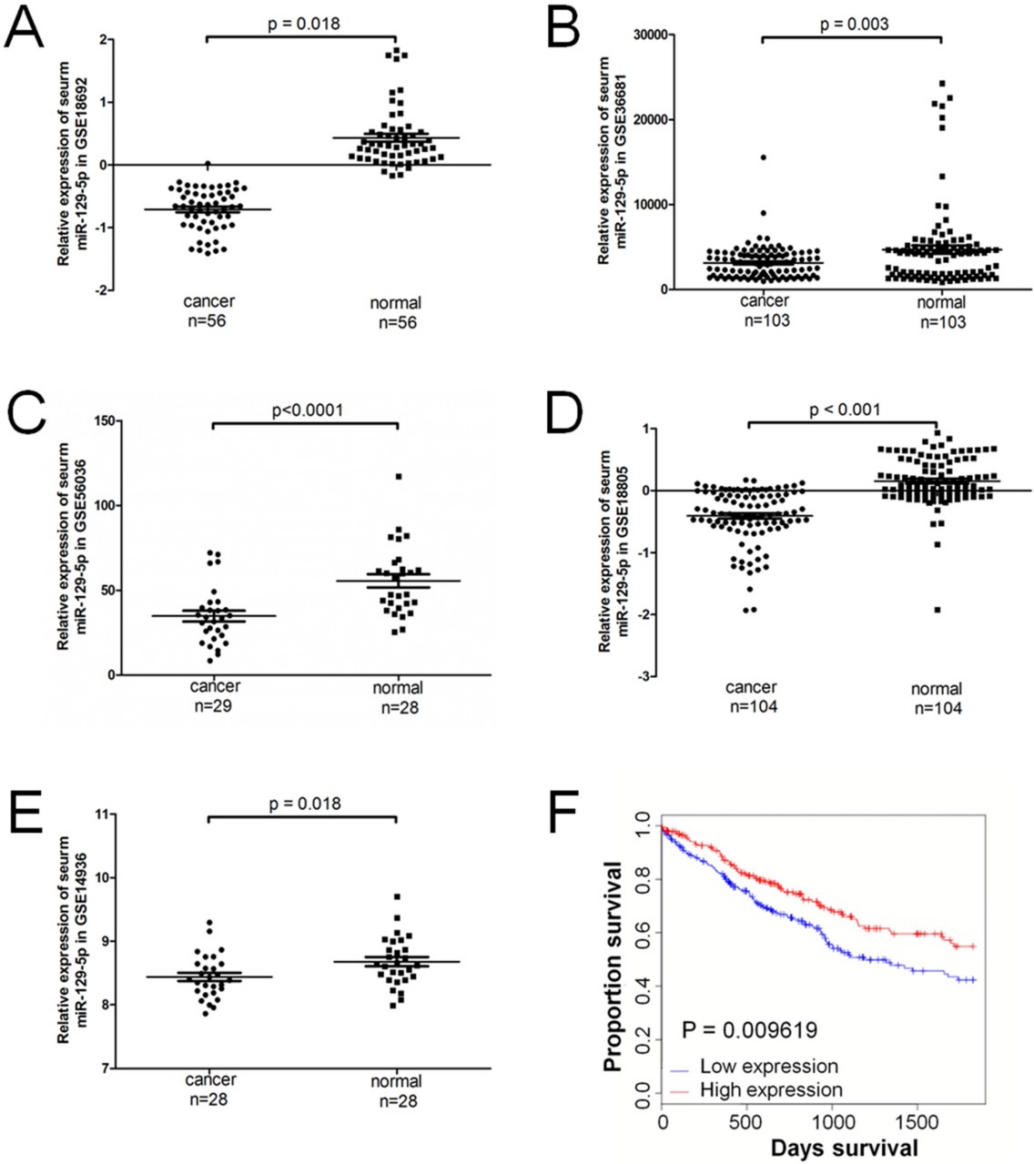
miR-129-5p is down-regulated in human lung cancer tissues and associated with lung cancer progression. **Note: (A-E)** Relative expression of miR-129-5p in lung cancer tissues and normal control tissues based on the array data retrieved from the GEO online database. **(F)** Kaplan-Meier survival analysis for the relationship between survival time and miR-129-5p signature in lung cancer was performed by using the online tool (http://kmplot.com/analysis/).

A total of 5 series (GSE) in the GEO database were extracted for the present study, which consisted of 320 lung cancer samples and 319 normal controls. The expression level of miR-129-5p was significantly downregulated in patients with lung cancer in all the 5 GEO datasets (**Figure 1A-1E**). Compared with the non-cancerous controls, the miR-129-5p lung cancer expression profiles were significantly downregulated in GSE18692, GSE36681, GSE56036, GSE18805 and GSE14936 (P<0.05). A significant correlation was found between the level of miR-129-5p and over survival of the patients with lung cancer (**Figure 1F**).

### Overexpression of miR-129-5p promotes VP16-induced lung cancer cell apoptosis

**Figure 2:**
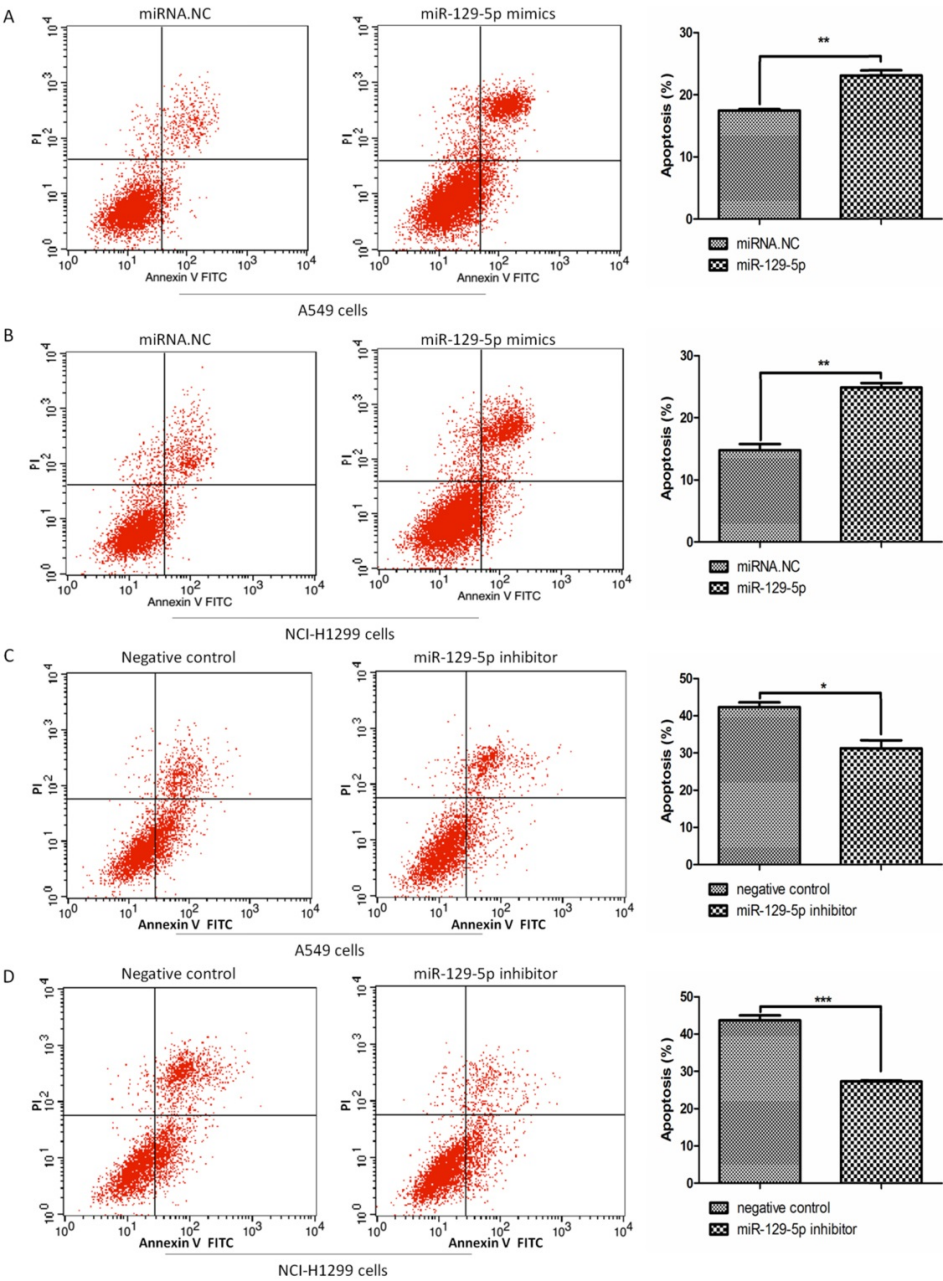
Overexpression of miR-129-5p promotes VP16-induced lung cancer cell apoptosis. **Note: (A)** Annexin-V FITC/PI assay was used to detect apoptotic cells in A549 cells treated with miR-129-5p mimic/NC (20nM). The rates of apoptosis in A549 cells were quantified (right panel). **(B)** Annexin-V FITC/PI assay was used to detect apoptotic cells in H1299 cells treated with miR-129-5p mimic/NC (20nM). The rates of apoptosis in H1299 cells were quantified (right panel). **(C)** Annexin-V FITC/PI assay was used to detect apoptotic cells in A549 cells treated with miR-129-5p inhibitor/NC (40nM). The rates of apoptosis in A549 cells were quantified (right panel). **(D)** Annexin-V FITC/PI assay was used to detect apoptotic cells in H1299 cells treated with miR-129-5p inhibitor/NC (40nM). The rates of apoptosis in H1299 cells were quantified (right panel).

To verify the effects of miR-129-5p on lung cancer cell apoptosis, we transfected A549 and H1299 cells with miR-129-5p mimics or miR-129-5p inhibitors, followed by the treatment of VP16 for 48 h. FCM was performed to detect the number of apoptotic cells including early and late apoptosis. The results indicated that overexpression of the miR-129-5p mimic markedly promoted VP16‑induced apoptosis (**Figure 2A** and **2B**). By contrast, overexpression of miR-129-5p inhibitor elicited the opposite effect, inhibiting VP16‑induced apoptosis (**Figure 2C** and **2D**). These results showed that overexpression of miR-129-5p increased the apoptosis of human lung cancer cells induced by the VP16 treatment.

### YWHAB is a direct downstream target of miR-129-5p

**Figure 3:**
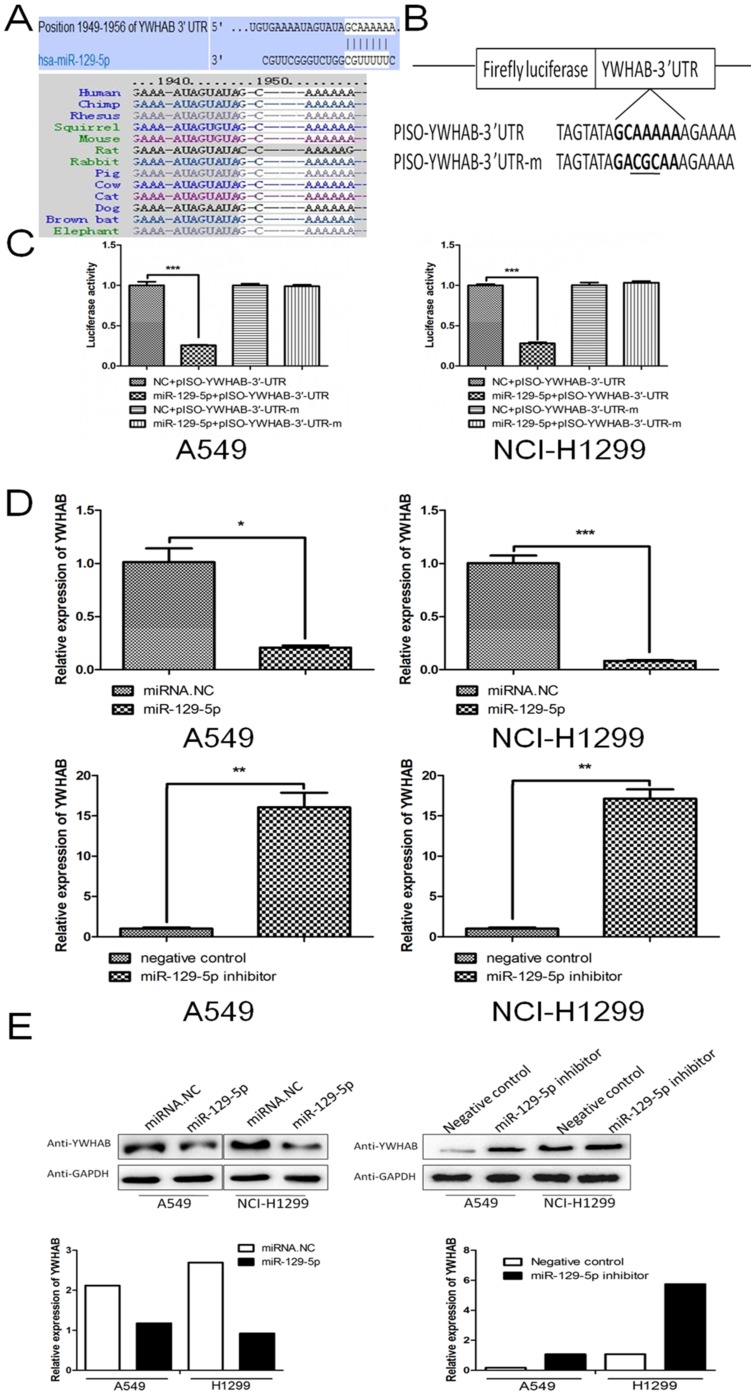
YWHAB is a direct downstream target of miR-129-5p. **Note: (A)** The potential interaction between miR-129-5p and putative binding sites in the YWHAB 3'-UTR predicted by TargetScan (upper panel). A highly-conserved miR-129-5p targeting sequence was predicted in the 3'-UTR of the YWHAB mRNA (bottom panel). **(B)** The sequences of the mutated YWHAB 3' UTR, in which several nucleotides within the miR-129-5p binding site were mutated. **(C)** The luciferase activity in the A549 and H1299 cells. Cells were cotransfected with miRNAs and luciferase report plasmids and the luciferase activities were detected in different groups. Each value is evaluated by the relative luciferase activity of firefly to renilla. **(D)** Effect of miR-129-5p on YWHAB mRNA level in the A549 and H1299 cells. After the cells were transfected, the YWHAB protein expression was detected by RT-PCR. β-actin was used for the internal control. **(E)** Effect of miR-129-5p on YWHAB protein level in the A549 and H1299 cells. After the cells were transfected, the YWHAB protein expression was detected by Western blot. GAPDH was used for the internal control. Relative levels of YWHAB were analyzed by quantification of the density of the bands with Image J software (bottom panel).

To reveal underlying mechanism by which miR-129-5p regulates cell apoptosis in lung cancer, we next searched for potential target genes of miR-129-5p in TargetScan (targetscan.org). The searching results predicted YWHAB as a candidate target of miR-129-5p. The 3'UTR of the YWHAB mRNA contained a complementary site for the seed region of miR-129-5p (**Figure 3A** upper panel). Homology search showed that the miR-129-5p targeting sequence at nucleotides 1949-1956 of the YWHAB-3'UTR was highly conserved among 13 species (**Figure 3A** bottom panel). To further validate whether miR-129-5p would bind directly to the 3'UTR of YWHAB, luciferase reporter vectors containing wide type (WT) 3'UTR or mutant 3'UTR target sequences were constructed. These constructs were cotransfected into A549 and H1299 cells with miR-129-5p mimic or its negative control for performing luciferase reporter assay (**Figure 3B**). Results demonstrated that miR-129-5p could repress the expression of reported gene containing WT 3'UTR but not the mutant 3'UTR (**Figure 3B**). The mRNA and protein levels of YWHAB in A549 and H1299 cells were also determined. The results showed that miR-129-5p reduced YWHAB expression at the mRNA and protein levels (**Figure 3D** and** 3E**). Collectively, these findings suggest that YWHAB is a direct downstream target of miR-129-5p.

### YWHAB is the key mediator of the effects of miR-129-5p in lung cancer cells

**Figure 4:**
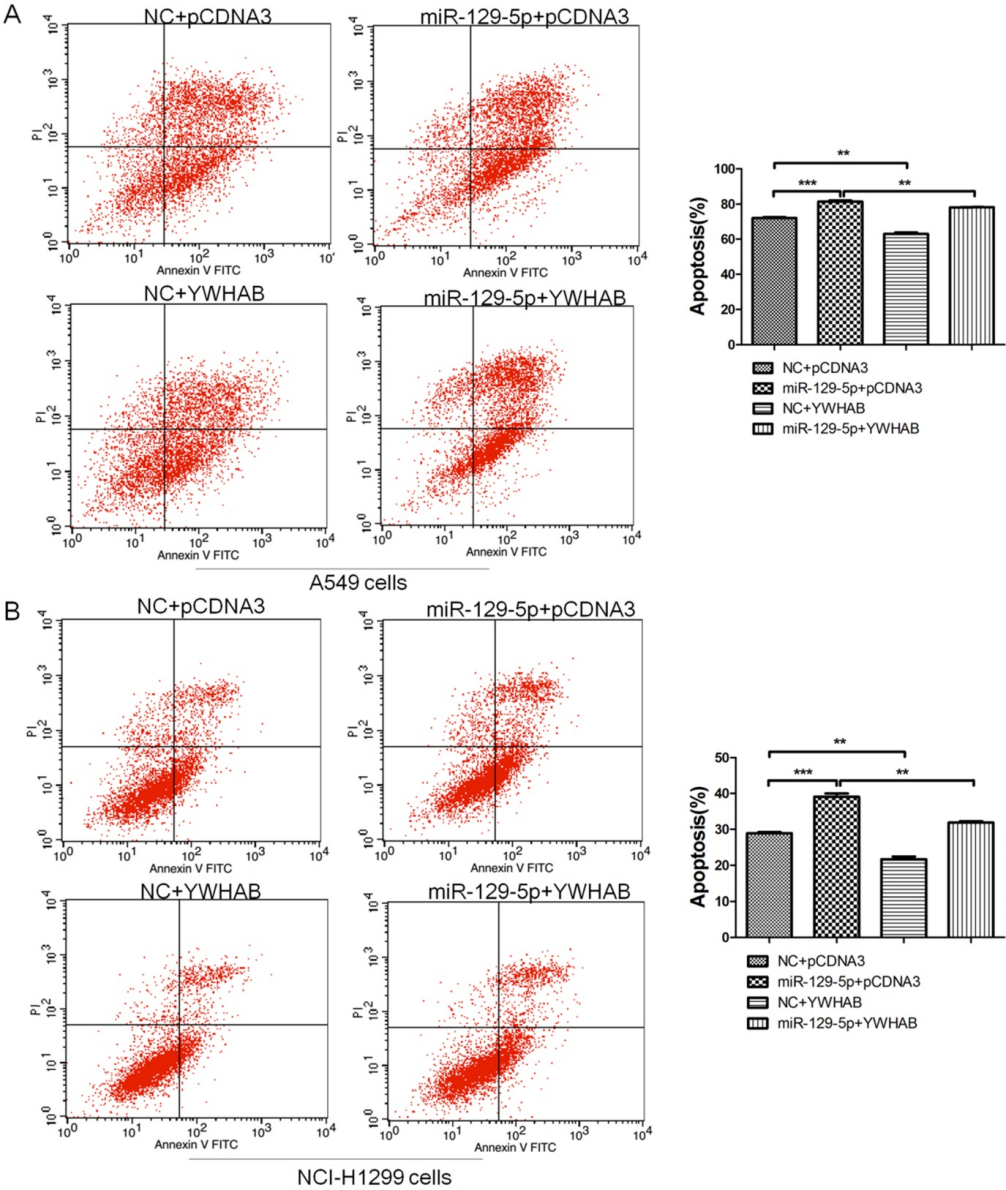
YWHAB is the key mediator of the effects of miR-129-5p in lung cancer cells. **Note: (A)** A549 cells were transfected with negative control (NC), miR-129-5p mimics and/or pCDNA3-YWHAB, and then cells were analyzed for apoptotic rate after staining with Annexin V-FITC and PI. Data represent means±S.D. from three independent experiments, ** P<0.01; *** P<0.001. **(B)** H1299 cells were transfected with negative control (NC), miR-129-5p mimics and/or pCDNA3-YWHAB, and then cells were analyzed for apoptotic rate after staining with Annexin V-FITC and PI. Data represent means±S.D. from three independent experiments, ** P<0.01; *** P<0.001.

To confirm that YWHAB is a functional target of miR-129-5p, A549 and H1299 cells were cotransfected with a negative control miRNA (NC) or miR-129-5p mimic, or a plasmid expressing YWHAB. As shown in **Figure 4A**, fewer apoptotic cells were detected after cotransfection with pCDNA3-YWHAB and miR-129-5p mimics compared to miR-129-5p alone. The same phenomenon was also observed in H1299 cells by FCM (**Figure 4B**). These results were consistent with the effects of miR-129-5p overexpression, indicating that miR-129-5p regulates lung cells apoptosis by directly targeting YWHAB.

### Elevated expression of YWHAB in lung cancer

**Figure 5:**
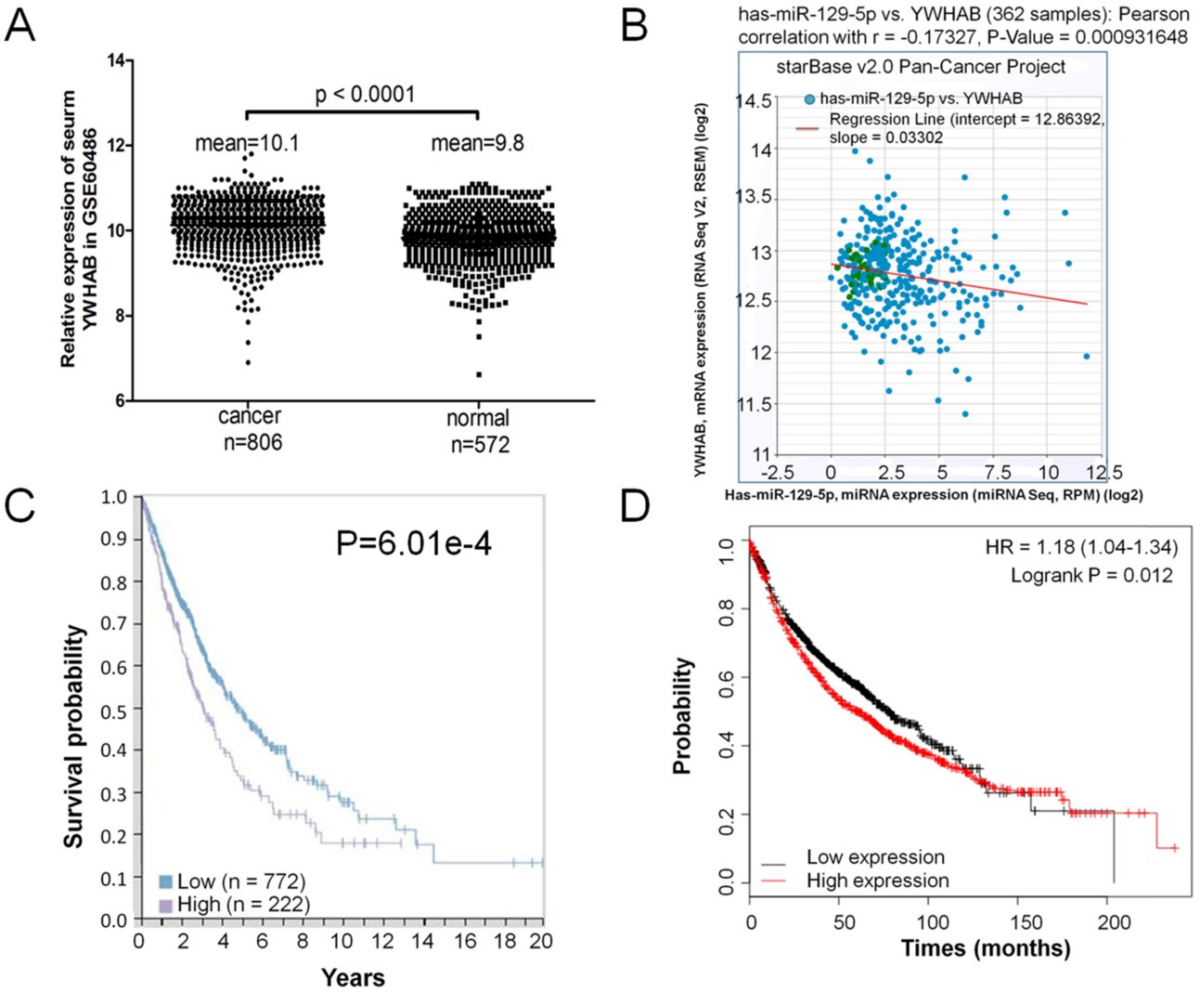
Elevated expression of YWHAB in lung cancer. **Note: (A)** RT-PCR analysis of GSE60486, YWHAB mRNA levels in patients' serum with lung cancer specimens (n=806) or normal control (n=572). **(B)** Analysis of correlation between YWHAB and miR-129-5p in lung cancer by using the online tool (http://starbase.sysu.edu.cn/index.php). **(C)** Kaplan-Meier survival analysis for the relationship between survival time and YWHAB signature in lung cancer was performed by using the online tool (www.proteinatlas.org/pathology). **(D)** Kaplan-Meier survival analysis for the relationship between survival time and YWHAB signature in lung cancer was performed by using the online tool (http://kmplot.com/analysis/).

A GEO database (GSE60486) was extracted for the present study, which consisted of 806 lung cancer samples and 572 normal controls. The expression level of YWHAB was significantly upregulated in patients' serum with lung cancer compared the normal control (**Figure 5A**). The correlation of miR-129-5p and YWHAB in lung cancer tissues was also determined. With the log10-transformed data, the Pearson correlation analysis showed a significant negative correlation between miR-129-5p and YWHAB (R=-0.173, p=0.0009) (**Figure 5B**). We then explored correlation analyses based on mRNA expression levels of YWHAB in lung cancer tissue and the clinical outcome (survival) of the patient using a recently published interactive open-access database (www.proteinatlas.org/pathology). As expected, increased expression of YWHAB is associated significantly with an unfavorable prognosis (survival) in patients with lung cancer (**Figure 5C**). We also performed Kaplan-Meier survival analysis of YWHAB with an online tool (http://kmplot.com/analysis/). The results showed that higher YWHAB expression was associated with a worse overall survival for patients with lung cancer (**Figure 5D**).

## Discussion

In this study, five microarray datasets from GEO public database was used to analysis expression of miR-129-5p in lung cancer. Our analysis of publically available databases revealed that miR-129-5p is significantly downregulated in tumor tissue compared with matched adjacent normal tissues. These findings point to an important role for changes in miR-129-5p expression during lung cancer progression.

Although many studies have explored the role of miR-129-5p in cancer 7, 27-29, there are few reports about the relationship between miR-129-5p expression and chemosensitivity of lung cancer. Lu et al. found that miR-129-5p sensitized Her-2-positive breast cancer to trastuzumab by downregulating RpS6 30. Ma et al. reported that miR-129-5p inhibits NSCLC stemness and chemoresistance through direct targeting of DLK1 31. Zeng et al. found that microRNA-129-5p suppresses Adriamycin resistance in breast cancer by targeting SOX2 32. These results suggested that miR-129-5p expression may have effects on the chemosensitivity of tumors. In the current study, we examined the effects of miR-129-5p expression on chemosensitivity of A549 cells and H1299 cells. The FCM assay showed that overexpression of miR-129-5p promoted the chemosensitivity of lung cancer cells. Moreover, decreased miR-129-5p level inhibited apoptosis of lung cancer cells after VP16 induction.

In conclusion, our results revealed that miR-129-5p is down-regulated in human lung cancer tissues and associated with lung cancer progression. Overexpression of miR-129-5p promotes VP16-induced lung cancer cell apoptosis. YWHAB is a direct downstream target of miR-129-5p and a key mediator of the effects of miR-129-5p in lung cancer cells. Higher YWHAB expression was associated with a worse overall survival for patients with lung cancer. Our findings provide a new role of miR-129-5p in lung cancer, and it may be considered as a potential novel target for therapeutic interventions in lung cancer. miR-129-5p maybe function as a tumor-suppressor in lung cancer and we could deliver it or over-express it in lung cancer cells to enhance lung cancer cell apoptosis. Ectopic expression of miR-129-5p and YWHAB were found in lung cancer tissue or patients' serum and both of them were closely related to the overall survival of patients, which could be used as biomarkers for monitoring the prognosis of lung cancer patients.

## Supplementary Material

Table S1.

## Abbreviations

GEO: Gene Expression Omnibus
YWHAB: 14-3-3 protein beta/alpha
NSCLC: non-small-cell lung cancer
UTR: Untranslated region
FCM: Flow cytometry
NC: Negative control
