# Supplementary material for: MiR-129-5p sensitization of lung cancer cells to etoposide-induced apoptosis by reducing YWHAB
Source: J Cancer. 2020 Jan 1;11(4):858–66. doi: 10.7150/jca.35410 (PMC6959023; doi:10.7150/jca.35410)
Supplement: Supplementary file 1 — Table S1. [file jcav11p0858s1.pdf]

Table S1. Primers used in this study

miRNA-129-5P-F	ACACTCCAGCTGGG CTTTTTGCGGTCTGG
miRNA-129-5P-R	CTCAACTGGTGTCGTGGAGTCGGCAATTCAGTTGAG GCAAGCCC
YWHAB-F	GTCACCAGGTCTCCCAAGTG
YWHAB-R	TTCTCCCCACTGCAGTGTTC
YWHAB-UTR-F	ttGAGCTCAGCAGAACTCTTACTGGTAGA
YWHAB-UTR-R	gcTCTAGAAAGACATACACAAATCTAGTACATCA
YWHAB-UTR-M-F	TGAAAATAGTATAGC GGCAAAGAAAAATCCCCG
YWHAB-UTR-M-R	CGGGGATTTTTCTTTGCCGCTATACTATTT TCA
U6-F	CTCGCTTCGGCAGCACA
U6-R	AACGCTTCACGAATTTGCGT
miRNA URP	TGGTGTCGTGGAGTCG
